# Lack of Influence of Vitamin D Receptor BsmI (rs1544410) Polymorphism on the Rate of Bone Loss in a Cohort of Postmenopausal Spanish Women Affected by Osteoporosis and Followed for Five Years

**DOI:** 10.1371/journal.pone.0138606

**Published:** 2015-09-22

**Authors:** Maria Pedrera-Canal, Jose M. Moran, Vicente Vera, Raul Roncero-Martin, Jesus M. Lavado-Garcia, Ignacio Aliaga, Juan D. Pedrera-Zamorano

**Affiliations:** Metabolic Bone Diseases Research Group, University of Extremadura, Nursing Department, Caceres, Spain; Universitat de Lleida-IRBLLEIDA, SPAIN

## Abstract

A longitudinal study was conducted to investigate the relation between a polymorphism in the vitamin D receptor (VDR) gene and changes in bone mineral density (BMD) and quantitative ultrasound of the phalanges (QUS) over a five-year period. The subjects were 456 postmenopausal women with osteoporosis undergoing treatment, aged 59.95±7.97 years (mean±standard deviation [SD]) at baseline. BMD was measured at the hips and lumbar spine by dual-energy X-ray absorptiometry, and QUS was measured by means of amplitude-dependent speed of sound (Ad-SoS) at the phalanges. Lifestyle information was obtained via a questionnaire. The genotype frequencies of the BsmI (rs1544410) gene polymorphism were 29.4%, 47.1%, and 23.5% for bb, Bb, and BB, respectively. After five years, BMD (annual change in %/year) at the femoral neck (FN) showed a significant modification based on the rs1544410 genotype (BB vs Bb); there was an overall decrease in bone mass (-0.70±2.79%/year; P = 0.025). An analysis of covariance with adjustments for age, weight, height, percentage of weight change per year, baseline BMD and calcium intake showed that the observed associations were no longer significant (P = 0.429). No significant associations were found between the QUS measurements and the rs1544410 genotype after the five-year period. Our study limitations includes lack of information about type and length of duration of the osteoporosis treatment. Our results indicate that rs1544410 polymorphisms do not account significantly for the changes in bone mass in Spanish women with osteoporosis undergoing treatment.

## Introduction

Osteoporosis is defined as a skeletal disorder characterized by loss of bone strength that predisposes to an increased risk of fracture, with bone strength primarily defined by the density and quality of bone [1]. Bone mineral density (BMD) is usually used as a measure of bone strength, and diagnoses of osteoporosis are based on its analysis [1].

BMD is influenced by genetic and environmental factors (such as physical activity, smoking, and diet). [2]. In particular, several twin and family studies have provided data that indicate that certain gene polymorphisms may play a major role in BMD, accounting for up to 50–80% of the inter-individual variation in bone mass [3–6].

Genes hypothesized to play a role in osteoporosis include those involved in bone formation and remodeling (e.g., LRP5), those involved in hormone signaling (e.g., VDR and ESR1), and those that encode bone structure proteins (e.g., COL1A1) [7,8]. However, studies of genetic associations still produce conflicting results, due at least partially to a lack of statistical power and different experimental approaches and study designs.

Since Morrison et al [9] reported a relationship between the VDR gene and BMD, discordant studies have been published; it is still not clear whether VDR genotypes influence bone mass accretion and/or postmenopausal bone loss [10–15]. Particularly, in Spanish population we have recently showed a lack of association of vitamin D receptor BsmI gene polymorphism (rs1544410) with bone mineral density in a cohort 210 unrelated healthy postmenopausal women [16].

Accordingly, the purpose of the present longitudinal study was to investigate the relation between changes in BMD over a five-year period and the common rs1544410 polymorphism in the VDR gene in postmenopausal Spanish women undergoing anti-osteoporotic therapy, after adjusting for potential confounding factors.

## Materials and Methods

This was an observational, longitudinal, prospective cohort study with a minimum follow-up period of five years. All the included women had osteoporosis and were undergoing conventional individualized treatments. The present study sample comprised 524 consecutive subjects included in the Caceres Database for the Diagnosis of Osteoporosis (CAFOR) study between 2009 and 2013. Subjects with complete data on BMD and QUS at the 5-year follow-up were included in the analysis. A total of 456 (87.02%) subjects were available for analysis. The study was performed in accordance with the Declaration of Helsinki and was approved by the Research Ethics Committee of the University of Extremadura. Written informed consent was obtained from all the subjects.

### Laboratory analysis

The following SNP was included: rs1544410. SNP was evaluated by allelic discrimination real-time PCR using a TaqMan probe (Applied Biosystems, Foster City, CA, USA). The PCR consisted of a hot start at 95°C for 10 minutes followed by 40 cycles of 94°C for 15 seconds and 60°C for 1 minute. Fluorescence detection occurred at a temperature of 60°C. All assays were performed in 10-μl reactions using TaqMan Genotyping Master Mix in 48-well plates on a StepOne® instrument (Applied Biosystems). Control samples representing all possible genotypes and a negative control were included in each reaction. The concordance of blinded quality control samples was 100%.

### Densitometric study

Densitometric measurements were performed to determine the BMD in the femoral neck (FN), femoral trochanter (FT), Ward’s triangle (WT) and the lumbar spine at the L2, L3, L4 and L2–L4 levels. Additionally, body weight and height were measured to calculate the body mass index (BMI). Densitometric tests were performed with the use of a NORLAND XR-800 (Norland Medical Systems, Inc.). BMD scores were expressed as grams per square centimeter.

### Quantitative ultrasound study

We assessed ultrasound bone status using an ultrasound device, model DBM Sonic 1200® (Emsor, S.A., Madrid, Spain), which measured the amplitude-dependent speed of sound (Ad-SoS) in meters per second. We measured the phalanges (II–V) of the nondominant hand and computed an average value. We achieved contact by means of standard ultrasound gel. Two 16-mm-diameter, 1.25-MHz transducers were assembled on a high-precision caliper that measured the distance between the probes. We positioned the probes on the mediolateral phalangeal surfaces using the phalanx head as a reference point. Positioning and repositioning the instrument was easy because it uses the prominences of the lower phalangeal epiphysis as a reference; the clip is placed just behind the prominences.

### Nutrients intake

Total dietary vitamin D, calcium and energy intake were assessed via validated frequency questionnaires, as previously described [17,18].

### Statistical analysis

Allelic and genotypic frequencies were estimated by gene counting, and the goodness of fit of the genotype distribution for Hardy-Weinberg equilibrium (HWE) was tested using a chi-square (χ^2^) test. Values of P>0.05 indicated HWE.

The statistical analysis of the results was performed with SPSS 20 for Windows. Normal distribution and homogeneity of variances were assessed using the Kolmogorov-Smirnov and Levene tests, respectively. An analysis of variance (ANOVA) followed by Bonferroni's post-hoc test was used to compare different genotypes in each SNP. An analysis of co-variance (ANCOVA) was used to compare the VDR genotypes adjusted for the co-variants age, BMI, years since menopause and daily calcium intake.

## Results and Discussion

Genotyping the rs1544410 polymorphism in the VDR gene of the 456 osteoporotic women showed that the distribution between the bb (n = 134), Bb (n = 215), and BB (n = 107) genotypes was in Hardy-Weinberg equilibrium.

The genotype distributions of the 456 women in relation to the other relevant characteristics are shown in Table 1. The groups were similar in terms of weight, BMI, calcium intake, QUS values and BMD values at the FN and L2–L4.

**Table 1:** Subject characteristics at Follow-Up visit by VDR rs1544410 Genotype.

	bb (n = 134)	Bb (n = 215)	BB (n = 107)	P-value
Follow-up time ± SD (years)	4,6 ± 0,7	4,6 ± 1,2	4,6 ± 1,0	0,949
Age ± SD (years)	64,4 ± 7,7	65,2 ± 8,0	63,4 ± 8,2	0,165
Height ± SD (m)	1,53 ± 0,06	1,53 ± 0,06	1,51 ± 0,07	0,079
Weight ± SD (kg)	64 ± 10,7	63,1 ± 10,8	62 ± 10,2	0,343
Annual percentage weight change ± SD	1,34 ± 4,27	0,42 ± 3,85	0,41 ± 3,98	0,083
BMI ± SD (kg/cm2)	27,23 ± 4,86	26,93 ± 5,14	27,13 ± 5,56	0,862
Energy intake ± SD (kcal)	2274,9 ± 759,4	2257,4 ± 691,7	2253,3 ± 664,0	0,967
Calcium intake ± SD (gr/day) (crude)	1193,5 ± 473,9	1249,1 ± 506,6	1156,9 ± 468,1	0,296
Calcium intake ± SD (gr/day) (energy adjusted)	1191,2 ± 41,1	1249,8 ± 32,7	1158,4 ± 46,1	0,069
Baseline FN BMD ± SD (g/cm2)	0,700 ± 0,092	0,713 ± 0,058	0,691 ± 0,103	0,096
Follow-up FN BMD ± SD (g/cm2)	0,685 ± 0,093	0,683 ± 0,093	0,689 ± 0,0952	0,844
Baseline TR BD ± (g/cm2)	0,557 ± 0,086	0,562 ± 0,084	0,541 ± 0,082	0,132
Follow-up TR BMD ± (g/cm2)	0,572 ± 0,097	0,567 ± 0,083	0,576 ± 0,084	0,653
Baseline L2-L4 BMD ± (g/cm2)	0,747 ± 0,070	0,737 ± 0,071	0,768 ± 0,069	0,409
Follow-up L2-L4 BMD ± (g/cm2)	0,759 ± 0,087	0,750 ± 0,089	0,755 ± 0,091	0,597
Baseline Ad-SoS (m/s)	2001,719 ± 68,418	2002,71 ± 74,896	2013,66 ± 77,127	0,386
Follow-up Ad-Sos (m/s)	1928,169 ± 100,260	1929,164 ± 100,027	1940,35 ± 114,861	0,609

The relationships between VDR rs1544410 genotype and BMD/QUS annual variation are shown in Table 2. A significant relationship between BMD variation at the FN and the rs1544410 genotype was observed: the BB group gained more BMD than the Bb group (Bonferroni post-test for ANOVA; P = 0.025 for Bb vs BB). However, this association did not remain significant after further adjustments for potential confounding factors (P = 0.429) (Table 2). The differences between genotypes at L2–L4 and for QUS measurements were not significant (Table 2).

**Table 2:** Changes of BMD and QUS in relation to VDR rs1544410 genotype (mean ± SD) Bonferroni post-ANOVA TEST.

Subject groups		bb (n = 134)	Bb (n = 215)	BB (n = 107)	P-value	bb vs Bb	bb vs BB	Bb vs BB
FN BMD change (%/y)	Unadjusted	-0,22 ± 3,17	-0,70 ± 2,79	0,26 ± 3,45	**0,027**	0,461	0,686	**0,025**
	Adjusted^a^	-0,33 ± 0,22	-0,48 ± 0,18	-0,027 ± 0,25	0,155	1	1	0,429
L2-L4 BMD change (%/y)	Unadjusted	-0,11 ± 2,52	-0,07 ± 2,92	-0,25 ± 2,48	0,859			
	Adjusted^a^	-0,09 ± 0,23	-0,11 ± 0,18	-0,14 ± 0,26	0,181			
Ad-SoS change (%/y)	Unadjusted	-0,70 ± 1,10	-0,70 ± 1,11	-0,75 ± 1,04	0,923			
	Adjusted^a^	-0,69 ± 0,09	-0,68 ± 0,07	-0,77 ± 0,11	0,527			

^a^ Adjusted for age, weight, height, percentage weight change per year, baseline BMD and calcium intake.

Multiple regression analyses were performed to evaluate the contributions of the genotypes for the rs1544410 VDR gene polymorphism. The results demonstrated that the annual changes in BMD (%) and QUS did not correlate with the number of B alleles. These findings indicate that the percentage change in BMD/QUS was independent of the patients’ alleles but was negatively modulated by age and baseline BMD (for FN), positively modulated by baseline BMD (for L2–L4) and calcium intake and, in the case of QUS determinations, negatively modulated by baseline Ad-SoS, age and weight (Table 3).

**Table 3:** Multiple regression models* examinig the influences of the rs1544410 polymorphism of the VDR gene on the percentage change in BMD at FN and L2-L4 and QUS at the phalanges (n = 432).

Variables	Variation explained (%)	β	95% CI for β	P-value
Dependent variable, annual change in FN BMD				
Age	9,8	-0,032	-0,062 to -0,002	0,039
Baseline FN BMD	55,4	-18,85	-21,47 to -16,23	<0,001
Dependent variable, annual change in L2-L4 BMD				
Baseline L2-L4 BMD	47,9	18,41	15,26 to 21,56	<0,001
Calcium intake	14,6	0,001	0,000 to 0,001	0,002
Dependent variable, annual change in Ad-SoS				
Baseline Ad-SoS	44,8	-0,007	-0,008 to -0,006	<0,001
Age	16,3	-0,20	-0,032 to -0,009	0,001
Weight	9,6	-0,009	-0,018 to 0,000	0,046

* From age, weight, height, weight change per year, baseline BMD, VDR rs1544410 genotype and calcium intake.

The purpose of our study was to investigate the possible effects of the rs1544410 VDR polymorphism on changes in BMD in postmenopausal osteoporotic women receiving treatment. We observed that the rs1544410 genotype of the VDR gene was not associated with the annual evolution of BMD and QUS in these patients.

Conflicting reports have been published with regard to the association of rate of bone loss with VDR genotypes [19–21]. We detected a modest association between the BB genotype (vs the Bb genotype) and the rate of bone loss at the FN, but not at the other skeletal sites measured. Adjustments for dietary, anthropometric and densitometric factors caused the association to cease to be significant. Due to the heterogeneity of gene-environmental interactions, the lack of appropriate adjustments is suggested to be one of the main causes of differences between studies that analyze associations between VDR genotypes and BMD.

Genetic association studies are well known for the poor replicability of the presented results [22]; it is always difficult to determine whether the presented associations are due to true associations or to a plethora of confounding factors, differences in methodology or patient recruitment designs [23]. Hence, common polymorphic variations within VDR genes have been associated with varying responses to anti-osteoporotic treatment [24,25]. To date, few studies have examined the possible effects of VDR genotype on BMD or bone loss in postmenopausal women under antiosteoporotic treatment; hence, the results are still conflicting. [26–28]. A study by Creatsa and colleagues [26] showed that postmenopausal women receiving alendronate had different BMD responses according to the VDR rs1544410 genotype. In fact, the BB genotype appeared to have the worst response at the lumbar spine, compared with patients with at least one b allele. Similarly, in a study of the effectiveness of antiresorptive treatment in postmenopausal women, treatment outcomes were found to be influenced by treatment arm, genotype at the rs1544410 variant and treatment arm × genotype interactions [29]. The same cohort in a previous study also reported a better response in women with the b allele [30,31]. However, Tofteng and colleagues [32] did not find differences between rs1544410 genotypes after five years of follow-up in a cohort of postmenopausal Danish women in the presence of a modifying effect that relied on non-genetic factors; our results are more in accordance with this study.

There have been only a handful of studies since the first report of associations between the VDR rs1544410 variant and postmenopausal women undergoing treatment, and as shown, even these relatively few studies have produced inconsistent findings. Hence, one of the main strengths of our study is that it adds to the limited core of knowledge in the area of associations between the VDR rs1544410 genotype and osteoporosis treatment in postmenopausal women. However, there are also limitations to our study. Because of the complexity of gene-gene interactions, it is possible that no single genotype-phenotype association is sufficiently strong to be evident; rather, a combination of genotypes may be required for a meaningful effect. Therefore, variations in BMD in postmenopausal osteoporotic women may be influenced by other genes that we did not investigate, including other variants of the VDR gene and collagen type I alpha 1 (COL1A1) and estrogen receptor polymorphisms. Differences in genetic background may also alter the outcome of association studies and complicate comparisons with previous studies. Major limitation of our study also includes the inability to provide information about type and length of duration of the osteoporosis treatment so we were unable to completely adjust our results for these factors in the statistical analysis.

Although it previously seemed that rs1544410 genotype might be involved in the individual response to osteoporosis treatment in postmenopausal women, our study indicates that the picture is still complicated: our results from a 5-year longitudinal study do not support the contention that the rate of bone loss is associated with the VDR polymorphism defined by the restriction enzyme rs1544410 in women undergoing osteoporosis treatment. Whether the presence of the b allele leads to a better response to treatment is uncertain; clearly, further work is required to fully assess the implications of treatment in these women.
